# Exploring the association of natriuretic peptides with QTc interval in hemodialysis patients

**DOI:** 10.1080/0886022X.2025.2460720

**Published:** 2025-02-17

**Authors:** Yoshihiro Matsumoto, Yasuo Mori, Shinji Kageyama, Kazuaki Yoshimura, Takao Saito, Risako Terada, Yohichi Nojima

**Affiliations:** ^a^ Nephrology and Dialysis, Shizuoka City Shizuoka Hospital, Shizuoka, Japan; ^b^ Hemodialysis, Shibukawa Clinic, Shizuoka, Japan; ^c^ Urology, Kageyama Clinic, Shizuoka, Japan; ^d^ Hemodialysis, Higashi-Shizuoka Jin Clinic, Shizuoka, Japan

**Keywords:** Cardiovascular disease, hemodialysis, QTc interval, N-terminal proBNP, sudden cardiac death

## Abstract

**Background:**

In patients undergoing hemodialysis (HD), cardiovascular (CV) disease, particularly sudden cardiac death (SCD), is a major cause of mortality. Independent predictors of SCD include a prolonged QT interval on electrocardiography (ECG) and elevated levels of natriuretic peptides (NPs). This study explores the association between the QTc interval and NPs in HD patients.

**Methods:**

This cross-sectional study involved 207 HD patients, having a heart rate of 57 to 103 bpm, displaying sinus rhythm and no extrasystoles in ECG reports. Before the 2nd HD of the week, we conducted ECG and blood tests for atrial NP (ANP), brain NP (BNP), and N-terminal proBNP (NT-proBNP). The heart rate-corrected QT (QTc) was calculated using Bazett formula. Our analysis focused on the association between QTc and each NP, along with evaluating clinically relevant variables related to the QTc interval.

**Results:**

Univariate analyses indicated robust correlations among the NPs, with each NP significantly associated with the QTc interval. Multiple regression analyses of the three NPs revealed that NT-proBNP demonstrated the strongest predictive ability for the QTc interval. Independent predictors of prolonged QTc included lower corrected calcium (cCa) levels (*p* = 0.001), lower potassium (K) levels (*p* < 0.001), and higher log NT-proBNP (*p* = 0.004).

**Conclusion:**

In HD patients, NT-proBNP shows a stronger link with the QTc interval than BNP or ANP. Integrating clinical management considering both QTc and log NT-proBNP levels might help reduce CV events. Additionally, vigilance regarding low K or cCa levels is recommended from the perspective of the QTc interval.

## Introduction

Patients with end-stage renal disease (ESRD) on dialysis are at a heightened risk of cardiovascular (CV) disease, with sudden cardiac death (SCD) being the leading cause of mortality [1]. Contrary to common assumptions, retrospective analyses revealed that 71% of dialysis patients experiencing SCD exhibit either normal left ventricular function or mild to moderate dysfunction [2]. Studies using implantable cardiac monitors in hemodialysis (HD) patients highlighted the prevalence of severe bradycardia with asystole in SCD cases [3,4], suggesting critical roles of dynamic electrical changes associated with profound myocardial alterations in this population. Recent multivariate analyses also linked bradyarrhythmia to prolonged heart rate-corrected QT (QTc) or PR intervals in HD patients [5].

A prolonged QT interval is recognized as an independent risk factor for SCD in older individuals [6] and heart failure patients [7]. Genovesi et al. demonstrated that prolonged QTc was linked to SCD and overall mortality in HD patients [8]. Our recent retrospective studies indicated that, compared to control individuals, HD patients had a longer QTc interval one year after HD initiation, with further prolongation associated with dialysis vintage [9]. These findings imply that clinical management focusing on the QTc interval could help reduce SCD risk in HD patients.

Interest has also increased in using natriuretic peptides (NPs) to prognosticate CV disease in ESRD. N-terminal pro-brain NP (NT-proBNP) was reported to offer crucial mortality risk insights in HD patients [10]. A recent meta-analysis identified ESRD-specific NT-proBNP and BNP thresholds linked to higher CV and all-cause mortality risks [11]. The Choices for Healthy Outcomes in Caring for ESRD study further emphasized the predictive power of elevated NT-proBNP levels in dialysis patients for SCD [12]. Additionally, atrial NP (ANP) and BNP have been shown to correlate with left atrial volume, reflecting diastolic dysfunction and volume overload in HD patients [13]. Elevated ANP levels also served as a strong predictor of long-term mortality across all stages of heart failure in patients not undergoing dialysis [14].

While the QTc interval or BNP/NT-proBNP level could be considered to independently predict prognosis in HD patients, it’s unclear whether there’s an association between the QTc interval and these NPs in these patients. In this study, we assessed the relationship between the QTc interval and each NP, as well as clinically relevant variables related to the QTc interval, in a population with a higher risk of SCD.

## Methods

### Study design

A multicenter cross-sectional study.

### Patient selection

Patients were selected from three outpatient HD clinics between August 2019 and February 2020. Approximately 450 patients were receiving at these clinics during the study period. Eligibility was determined by reviewing medical records and ECG from the previous 3 months. Patients who were 30 years or older and had been undergoing 4-h long HD three times a week for at least 5 months were included in the study. The exclusion criteria were hypotension, hepatic failure, active malignancy, or any life-threatening disease other than ESRD. Any patients who had a heart rate <57 beats per minute (bpm) or >103 bpm, any rhythm other than sinus, or any instances of extrasystoles in their ECG reports were excluded for accurate QTc assessment, as in a previous paper [9].

This study received approval from the Ethics Committee of Shibukawa Clinic (Approval Number: 004) and adhered to the principles of the Declaration of Helsinki, including voluntary participation, anonymity, confidentiality, consideration of potential harm, and results communication. Written informed consent, emphasizing voluntary participation, was obtained from all participants. The collection of their blood samples involved minimal volume, ensuring their well-being. The research was conducted in strict accordance with established ethical procedures to safeguard participant rights and privacy.

### Data collection

In all three HD clinics, pre- and post-dialysis blood samples are routinely collected on the 1^st^ HD day of the week (Monday or Tuesday) every month, and ECG and chest radiography are performed before the HD session on the same day as blood tests at least every 3 to 6 months. However, for the current study, these examinations were performed on the 2^nd^ HD day of the week (Wednesday or Thursday) according to the standards of advanced dialysis in other countries. We obtained the following variables from the medical records on the same day: hemoglobin (g/dL), albumin (g/dL), urea (mg/dL), total calcium (tCa, mg/dL), phosphorus (mg/dL), potassium (K, mEq/L), magnesium (mg/dL) and glucose (mg/dL). Albumin-corrected serum calcium was calculated using the following equation: corrected calcium (cCa, mg/dL) = 0.8 × (4 - measured serum albumin [g/dL]) + measured tCa (mg/dL) [15]. Dialysate calcium (dCa) concentration (mEq/L), post-dialysis body weight (kg), intradialytic weight gain percentage (IDWG%), blood pressure (mmHg), cardiothoracic ratio (%) on chest radiography were recorded. IDWG% was calculated as (IDWG/Weight after last dialysis) × 100. Information on medications, including angiotensin-converting enzyme inhibitors (ACEIs), angiotensin receptor blockers (ARBs), beta-blockers, antiarrhythmic drugs, and those with known or potential QT interval prolongation (assessed using the CredibleMeds QTc Prolongation Database: https://crediblemeds.org), was collected. None of the patients received the combination drug sacubitril/valsartan, which affects the degradation of NPs, because it was unavailable. Medical histories of cardiac surgery, percutaneous coronary intervention (PCI), and implantation of a pacemaker or defibrillator were also documented.

### Measurement of NPs

Blood samples for measurement of ANP, BNP, and NT-proBNP were collected immediately before the HD session after ECG examination on the 2^nd^ HD day of the week. NP levels were assessed by the Special Reference Laboratory (Tokyo, Japan). ANP and BNP levels were measured using chemiluminescent enzyme immunoassay, and NT-proBNP levels were measured by electrochemiluminescence immunoassay. The upper limits of the reference range for ANP, BNP, and NT-proBNP were 43.0 pg/mL, 18.4 pg/mL, and 125 pg/mL, respectively.

### Evaluation of the QTc interval in ECGs

QTc interval was evaluated as described previously [9]. Patients were subjected to a 12-lead ECG at rest employing devices manufactured by Fukuda Denshi (Tokyo, Japan) or Nihon Kohden (Tokyo, Japan). An automated algorithm was used to assess QT and RR intervals. Both companies employed distinct programs to gauge the QT interval and employed different methodologies to calculate the QTc interval (Fukuda Denshi used Bazett formula, while Nihon Kohden used ECAPS12 formula). In order to unify the formula for determining the QTc interval, we recalculated the results from the Nihon Kohden device using Bazett formula (QTc = QT/√RR) based on the corresponding QT and RR intervals.

### Statistical analyses

Data are expressed as mean ± standard deviation or median (25^th^–75^th^ percentile) for continuous variables, or as percentage for categorical variables. All analyses were performed using DANS version 7.4 (Sugimoto Data Analysis Service, Nagoya, Japan).

Univariable analyses were used to identify factors related to the QTc interval. The unpaired t-test or one-way layout analysis of variance was applied for categorical variables, and linear regression analysis was applied for continuous variables. Multivariable regression analyses were performed to determine the effect of the three NPs on the QTc interval. Because the correlation coefficient matrix for NPs revealed that each NP had a strong correlation with the other NPs, the NP with the strongest association with the QTc interval was selected for further analyses. Multivariable regression analyses were also conducted to evaluate the association between the QTc interval and 13 variables (sex, age, principal cause of ESRD, dialysis vintage, medical history of PCI or cardiac surgery, weight, IDWG%, systolic blood pressure, cardiothoracic ratio, albumin, serum cCa, serum K, and NP). These variables were selected based on the difference in average, regression coefficient, and *p* value in the univariable analyses. For the multivariable analysis, the principal cause of ESRD was divided into two categories for accuracy: diabetes and others. A *p* value <0.05 was considered statistically significant.

## Results

### Patient enrollment and characteristics

Patient enrollment was conducted individually at each clinic, with medical staff identifying eligible patients based on predefined inclusion and exclusion criteria and obtaining informed consent. The process of obtaining informed consent presented certain challenges, as medical staff were occasionally hesitant to approach patients, particularly when they anticipated difficulties explaining the study plan or engaging with patients who had complex personalities or were less receptive. Of the approximately 450 patients receiving dialysis at the participating clinics, 232 were initially recruited and scheduled for ECG and blood examinations. However, 15 patients were excluded due to hospitalization or missing key data, and an additional 10 patients were excluded based on exclusion criteria related to the ECG performed immediately before the blood examination. Consequently, a total of 207 patients were ultimately enrolled in the study.

The clinical and demographic characteristics are summarized in Table 1. Of the enrolled patients, 63% were male. The mean age was 68.6 years, and the average dialysis vintage was 6.5 years. Diabetes was the principal cause of HD in 47% of patients, and 15% had a medical history of PCI or cardiac surgery. Notably, 10% of patients were taking medications with a known or possible risk of QT interval prolongation. Dialysate bathes contained 2.0 mEq/L K+, 2.5 or 3.0 mEq/L Ca++, and 1.0 mEq/L Mg++.

**Table 1.** Baseline characteristics of study populations.

Characteristic	Value
Male／Female	130／77
Age, yr	68.6 ± 12.0
Principal cause of ESRD	
Diabetes	98 (47.3)
Hypertension	12 (5.8)
Glomerulonephritis	41(19.8)
Polycystic kidney disease	11 (5.3)
Other	45 (21.7)
Duration on dialysis, yr	6.5 ± 6.2
History of PCI or cardiac surgery	32 (15.4)
Medication	
ACEI, ARB, or both	102 (49.3)
Beta-blocker	52 (25.1)
Ca-blocker	123 (59.4)
Digitalis	0 (0.0)
Other anti-arrhythmic drugs	3 (1.4)
QT-prolonging drugs	21 (10.1)
Weight, kg (after dialysis)	57 ± 13
Intradialytic weight gain percentage, %	3.1 ± 1.3
Blood pressure, mmHg	
Systolic	152 ± 26
Diastolic	77 ± 13
Cardiothoracic ratio, %	51 ± 5
Urea reduction ratio, %	71 ± 6
Dialysate calcium concentration	
2.5 mEq/L	155 (74.9)
3.0 mEq/L	52 (25.1)
Laboratory data (before dialysis)	
Hemoglobin, g/dL	10.9 ± 1.2
Albumin, g/dL	3.6 ± 0.3
Urea, mg/dL	53.2 ± 14.4
Corrected calcium, mg/dL	8.9 ± 0.5
Phosphorus, mg/dL	5.3 ± 1.1
Potassium, mEq/L	4.7 ± 0.7
Magnesium, mg/dL	2.4 ± 0.3
Glucose, mg/dL	136 ± 55
Natriuretic peptides	
ANP, pg/mL	127 (99–235)
BNP, pg/mL	179 (79–422)
NT-proBNP, pg/mL	4,896 (1,715–9,923)

Categorical data are shown as n (percentage). Continuous data are presented as mean ± standard deviation or median (25th–75th percentile). ESRD: end-stage renal disease; PCI: percutaneous coronary intervention; ACEI: angiotensin-converting enzyme inhibitor; ARB: angiotensin receptor blocker; ANP: atrial natriuretic peptide; BNP: brain natriuretic peptide; NT-proBNP: N-terminal proBNP.

### Prolonged QTc interval and its predictors other than NPs

The results of the univariable analyses are presented in Table 2. The principal cause of ESRD was found to be associated with the QTc interval (*p* = 0.049, by one-way layout analysis of variance). A medical history of PCI or cardiac surgery was also linked to longer QTc intervals (*p* = 0.007, unpaired t-test). The QTc interval demonstrated a direct relationship with age (*p* = 0.016) and cardiothoracic ratio (*p* < 0.001), and an inverse relationship with cCa (*p* < 0.001) and K (*p* < 0.001). The variables of sex, dialysis vintage, use of ACEIs/ARBs, beta-blockers, or QT-prolonging drugs, along with weight, IDWG%, blood pressure, dCa concentration, hemoglobin, albumin, urea, and phosphate did not exhibit statistically significant associations with the QTc interval. The association of the QTc interval with glucose and magnesium could not be evaluated due to a lack of data.

**Table 2.** Univariable analyses of various variables on QTc interval.

Variables	QTc	Regression line	*p*-Value
Sex			0.106
Male	448		
Female	453		
Age^a^, yr		*y* = 427 + 0.333x	0.016
Principal cause of ESRD^a^			0.049
Diabetes	454		
Hypertension	440		
Glomerulonephritis	447		
Polycystic kidney disease	438		
Other	448		
Duration on dialysis, yr		*y* = 451 − 0.223x	0.406
History of PCI or Cardiac surgery^a^			0.007
Yes	460		
No	448		
Medication of ACEI, ARB, or both			0.785
Yes	450		
No	449		
Medication of beta-blocker			0.265
Yes	453		
No	449		
Medication of QT-prolonging drugs			0.203
Yes	456		
No	449		
Weight, kg (after dialysis)		*y* = 463 − 0.241x	0.051
IDWG%		*y* = 443 + 2.11x	0.091
Blood pressure, mmHg			
Systolic		*y* = 432 + 0.114x	0.079
Diastolic		*y* = 441 + 0.105x	0.426
Dialysate calcium concentration			0.082
2.5 mEq/L	452		
3.0 mEq/L	444		
Cardiothoracic ratio^a^, %		*y* = 393 + 1.12x	<0.001
Hemoglobin, g/dL		*y* = 464 − 1.35x	0.329
Albumin, g/dL		*y* = 461 − 2.99x	0.587
Urea, g/dL		*y* = 459 − 0.173x	0.134
Corrected calcium^a^, mg/dL		*y* = 560 − 12.3x	<0.001
Phosphorus, mg/dL		*y* = 461 − 2.11x	0.175
Potassium^a^, mEq/L		*y* = 490 − 8.64x	<0.001
Natriuretic peptides			
Log ANP^a^		*y* = 419 + 14.2x	0.009
Log BNP^a^		*y* = 430 + 8.83x	0.005
Log NT-proBNP^a^		*y* = 399 + 14.0x	<0.001

Values are means. Unpaired t-test or one-way layout analysis of variance was applied for categorical variables. Linear regression analysis was applied for continuous variables. QTc: heart rate-corrected QT; ESRD: end-stage renal disease; PCI: percutaneous coronary intervention; ACEI: angiotensin-converting enzyme inhibitor; ARB: angiotensin receptor blocker; IDWG%: intradialytic weight gain percentage; ANP: atrial natriuretic peptide; BNP: brain natriuretic peptide; NT-proBNP: N-terminal proBNP.

^a^Significant associations (*p* < 0.05).

### Relationship between the QTc interval and each NP and between NPs by univariate analyses

**Figure 1.**
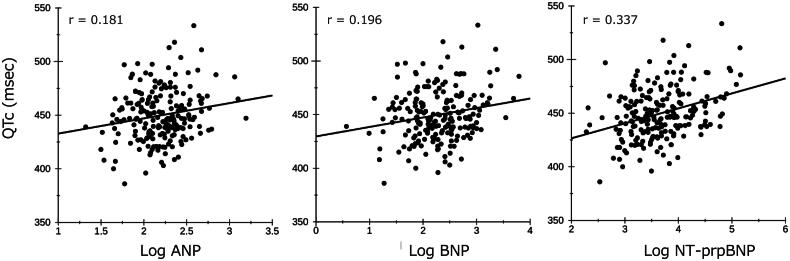
Correlation between logarithmic NPs and QTc interval. QTc: heart rate-corrected QT; NT-proBNP: N-terminal pro-brain natriuretic peptide.

The results of the univariable analyses assessing the relationship of NPs with the QTc interval are shown at the bottom of Table 2 and Figure 1. Log ANP, log BNP, and log NT-proBNP were all significantly positively correlated with the QTc interval (*p* = 0.009, *p* = 0.005, and *p* < 0.001, respectively). Table 3 presents a correlation coefficient matrix of the QTc interval and the three NPs. Each NP had a strong correlation with the other NPs, suggesting their close interrelationship.

**Table 3.** Correlation coefficient matrix of three NPs and QTc.

	Log ANP	Log BNP	Log NT-proBNP	QTc
Log ANP	1	0.846	0.750	0.181
Log BNP	0.846	1	0.871	0.196
Log NT-proBNP	0.750	0.871	1	0.337
QTc	0.181	0.196	0.337	1

NPs: natriuretic peptides; QTc: heart rate-corrected QT; ANP: atrial NP; BNP: brain NP; NT-proBNP: N-terminal proBNP.

### Multivariable regression analyses of the three NPs on QTc

Prior to conducting multiple regression analyses of selected variables including NP, we initially performed separate multiple regression analyses of the three NPs to identify the NP most significantly associated with the QTc interval. As shown in Table 4, the standard partial regression coefficient of log ANP had a small absolute value. Surprisingly, the partial regression coefficient of log BNP was negative (Table 4), in contrast to the positive simple correlation coefficient seen in Table 2. Consequently, among the three NPs, NT-proBNP exhibited the most substantial influence on the QTc interval.

**Table 4.** Multiple regression analyses of logarithmic NPs on QTc interval.

Covariates	Average	Partial regression coefficient	Standard partial regression coefficient	Variance Inflation Factor	Adjusted *p*-value
Log ANP	2.19	1.68	0.0213	3.53	0.861
Log BNPa	2.28	−18.9	−0.420	6.41	0.011
Log NT-proBNPa	3.66	28.5	0.686	4.15	<0.001

NPs: natriuretic peptides; QTc: heart rate-corrected QT; ANP: atrial NP; BNP: brain NP; NT-proBNP: N-terminal proBNP.

Multiple coefficient of determination is 0.153. ^a^Significant predictors (*p* < 0.05).

### Multivariable regression analyses of log NT-proBNP and other selected variables on QTc

We included log NT-proBNP and 12 other variables in the multivariable analysis based on the results of the univariable analyses. We included only cCa and not dCa because the simple correlation coefficient between the two was 0.284. Multiple regression analyses of the 13 variables showed that lower cCa levels (*p* = 0.001), lower K levels (*p* < 0.001), and higher log NT-proBNP levels (*p* = 0.004) were independent predictors of QTc interval prolongation, whereas sex, age, principal cause of ESRD, dialysis vintage, history of PCI or cardiac surgery, weight, IDWG%, systolic blood pressure, cardiothoracic ratio, and albumin levels were not associated with the QTc interval in the multivariable analysis (Table 5).

**Table 5.** Multivariable regression analyses of selected variables including log NT-proBNP on QTc interval.

Covariates	Average	Partial regression coefficient	Standard partial regression coefficient	Variance Inflation Factor	Adjusted *p*-value
Sex (1: Female, 0: Male,)	0.372	5.15	0.105	1.48	0.160
Age	68.6	0.142	0.0713	1.66	0.365
Principal cause of ESRD (1: Diabetes, 0: Others)	0.473	5.98	0.125	1.21	0.063
Duration on dialysis	6.5	−0.274	−0.0711	1.09	0.266
History of PCI or cardiac surgery (1: Yes, 0: No)	0.155	7.28	0.111	1.15	0.092
Weight	56.8	0.159	0.873	2.00	0.312
IDWG%	3.1	1.12	0.0624	1.19	0.350
Systolic blood pressure	152	−0.0204	−0.219	1.24	0.747
Cardiothoracic ratio	51.2	0.404	0.0898	1.57	0.241
Albumin	3.63	2.77	0.0353	1.28	0.609
Corrected calcium^a^	8.93	−9.17	−0.210	1.11	0.001
Potassium^a^	4.69	−8.77	−0.247	1.07	<0.001
Log NT-proBNP^a^	3.66	9.55	0.230	1.69	0.004

log NT-proBNP: logarithm of N-terminal pro-brain natriuretic peptide; QTc: heart rate-corrected QT; ESRD: end-stage renal disease; PCI: percutaneous coronary intervention; IDWG%: intradialytic weight gain percentage.

Multiple coefficient of determination is 0.283. ^a^Significant predictors (*p* < 0.05).

**Figure 2.**
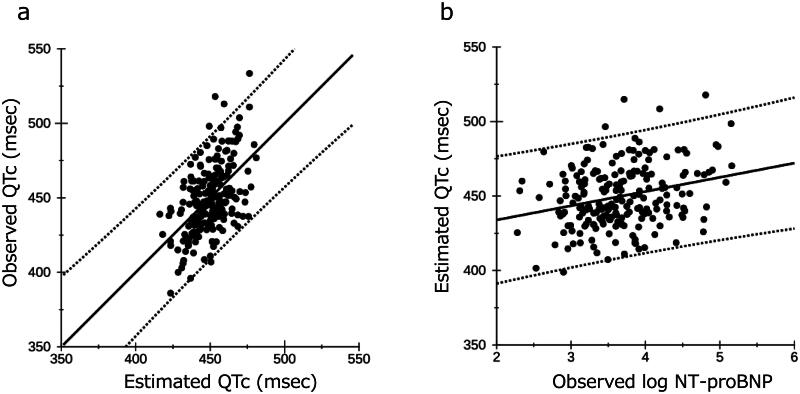
The associations between the estimated QTc and the observed QTc interval (a), and the observed log NT-proBNP and the estimated QTc (b). Both were obtained through a multiple regression equation. Multiple coefficient of determination is 0.283. The dotted line indicates the 95% prediction limit. QTc: heart rate-corrected QT; NT-proBNP: N-terminal pro-brain natriuretic peptide.

With a multiple coefficient of determination (R^2^) of 0.283, the QTc can be partially estimated from the observed 13 variables. Figure 2a shows the association between the observed QTc interval and the estimated QTc obtained through a multiple regression equation. In Figure 2b, the partial regression line represents the relationship between the observed log NT-proBNP and the estimated QTc, with all other variables held at their average values. The formula for this line is as follows:

Estimated QTc = 483.87 + 5.15 × 0.372 (mean sex) + 0.142 × 68.6 (mean age) + 5.98 × 0.473 (mean cause) − 0.274 × 6.5 (mean duration on dialysis) + 7.28 × 0.155 (mean history) + 0.159 × 56.8 (mean weight) + 1.12 × 3.1 (mean IDWG%) − 0.0204 × 152 (mean systolic blood pressure) + 0.404 × 51.2 (mean cardiothoracic ratio) + 2.77 × 3.63 (mean albumin) − 9.17 × 8.93 (mean cCa) − 8.77 × 4.69 (mean K) + 9.55 × log NT-proBNP = 414.8 + 9.55 × log NT-proBNP.

This formula indicates that a tenfold increase in NT-proBNP levels is associated with an estimated 10 ms prolongation of the QTc interval.

## Discussion

In the present study, we examined which NP had the most significant influence on the QTc interval and found that log NT-proBNP was the most efficient independent predictor of QTc interval prolongation. The burden of CV disease in patients undergoing dialysis is substantial because CV disease is both a significant cause and a consequence of ESRD. Effective CV risk stratification is a decisive aspect of managing dialysis patients, as it enables early identification of high-risk patients and optimized therapeutic interventions. Both the QTc interval, which is an ECG metric, and BNP or NT-proBNP, which are circulating cardiac biomarkers, can reflect pathophysiologic processes and are associated with clinical CV disease in dialysis patients [8,10,11]. Since they can independently predict CV death events in dialysis patients [10,12,16–18], incorporating these parameters with standard risk factors may enhance the accuracy of predicting cardiac death events among patients undergoing dialysis.

The QT interval on the surface ECG, representing the time from the onset of ventricular depolarization to the completion of repolarization, serves as one of the parameters for evaluating autonomic dysfunction. Just as decreased K and cCa in our dialysis patients were associated with a prolonged QTc interval, other factors, such as electrolyte balance, contribute to this parameter [19]; therefore, the QTc interval does not solely indicate the state of the autonomic system, but autonomic dysfunction is a significant contributor [20]. Since a prolonged QTc interval [21] and QT dispersion [22] have been shown to be predictors of SCD in patients with heart failure, autonomic dysfunction could be an important and potentially modifiable component of the process of heart failure in dialysis patients.

BNP has been used as a biomarker of heart failure in patients with and without CKD. During BNP secretion from cardiac myocytes, pro-BNP is cleaved to form a biologically active fragment (BNP) and an inactive N-terminal fragment (NT-proBNP). Transcription of *BNP* is induced by several pathophysiological stimuli, including mechanical myocardial stretch [23,24] or strain [25], ischemia [26], pro-inflammatory cytokines [27], vasoactive factors such as angiotensin II [28], and sympathetic overactivity [29]. As these stimuli have also been implicated in the genesis and progression of cardiomyopathy in patients with CKD [30], BNP/NT-proBNP may serve as crucial biomarkers for assessing cardiac risk in the dialysis population.

Considering that two markers of different origins, QTc and NT-proBNP, may be influenced by autonomic factors (sympathetic imbalance), our finding of the independent relationship between QTc interval prolongation and log NT-proBNP levels is plausible. According to a recent review [31], heart failure is associated with structural and electrical cardiac remodeling that predisposes the failing heart to arrhythmogenesis and SCD. This remodeling process is largely affected by sustained alterations in neurohormonal signaling, including the sympathetic nervous system and the renin-angiotensin-aldosterone system (RAAS) [32]. Furthermore, the interaction between the autonomic system and RAAS is intricately linked [20]. If neurohormonal dysfunction plays a role in arrhythmogenesis and SCD in HD patients, aldosterone receptor blockade might modify the CV risk, as the Randomized Aldactone Evaluation Study [33] and Dialysis Outcomes Heart Failure Aldactone Study [34] previously reported, and the CV risk may be controlled by NT-proBNP and QTc-driven management strategies.

While these neurohormonal mechanisms provide a possible explanation, statistical considerations and methodological limitations should be addressed. The opposing regression coefficients for log BNP and QTc interval in univariable versus multivariable analyses are noteworthy. In the multivariable analysis, the log BNP coefficient was negative (Table 4), whereas the simple correlation was positive (Table 2). This difference may reflect an influence from other variables, especially log NT-proBNP, as indicated by the high variance inflation factor (6.41; Table 4). Furthermore, assay-related limitations may affect the observed associations. Current immunoassay systems for BNP measurement also detect uncleaved proBNP, as the anti-BNP antibody cross-reacts with proBNP [35]. Consequently, the BNP levels in our study reflect a combination of BNP and proBNP, whereas NT-proBNP may provide a more precise measure of BNP transcription activity. Indeed, NT-proBNP exhibited a stronger association with the QTc interval compared to BNP, underscoring its potential importance in cardiological management.

We also found that lower K and cCa levels were independent predictors of QTc interval prolongation. In the Predictors of Arrhythmic and Cardiovascular Risk in End-Stage Renal Disease (PACE) study, lower ionized Ca (iCa) and K levels were associated with longer QTc intervals independent of potential confounders, whereas cCa was not significantly associated with the QTc interval [36]. In the PACE study, the QTc interval was categorized into two sex-specific groups as either ‘normal’ or ‘prolonged’; however, the chosen cutoff points were somewhat arbitrary [6,37]. Our analysis involved QTc interval data as a continuous variable, potentially accounting for the variations in results. Nevertheless, serum iCa could be utilized to stratify prolonged QTc and its potential risk for arrhythmia and SCD because hidden hypocalcemia (low iCa despite normal or high cCa) is a strong predictor of death and CV events [38].

This study has several important limitations. First, the cross-sectional design and smaller sample size of this small multicenter study limit the generalizability of our findings and preclude analysis of longitudinal trends in QTc interval and NT-proBNP levels. This design also does not allow for determination of temporal relationships, making it impossible to establish causation between NT-proBNP levels and QTc interval. Second, certain potential confounders were not included in our analysis. We lacked data on serum magnesium and glucose levels, which are known to influence QTc variability, as well as key medical history details (e.g., myocardial infarction, heart failure) and physiological measures like left ventricular ejection fraction. Given the R^2^ of 0.283 in our model, it is likely that including these variables would contribute further to explaining QTc variance. Lastly, while the observed associations between QTc and Log NT-proBNP in dialysis patients are noteworthy, they should be interpreted strictly as associative findings. The cross-sectional nature of our study precludes conclusions about causation, directionality, or underlying mechanisms. Furthermore, the use of QTc and NT-proBNP as risk stratification tools specifically in this population remains an area for future validation studies.

In conclusion, our study demonstrates that higher log NT-proBNP, lower K levels, and lower cCa levels are independent predictors of QTc interval prolongation. Our hope is that this work will inspire new research into NT-proBNP and QTc-driven approaches to stratify CV risk, including SCD risk, with higher accuracy. Additionally, we expect that lower K and cCa levels will be paid more attention in clinical dialysis with regard to their impact on the QTc interval.

## Data Availability

All data generated or analyzed during the study are included in this article. Further enquiries can be directed to the corresponding author.
